# Effect of Size on Magnetic Polyelectrolyte Microcapsules Behavior: Biodistribution, Circulation Time, Interactions with Blood Cells and Immune System

**DOI:** 10.3390/pharmaceutics13122147

**Published:** 2021-12-14

**Authors:** Roman Verkhovskii, Alexey Ermakov, Olga Sindeeva, Ekaterina Prikhozhdenko, Anastasiia Kozlova, Oleg Grishin, Mikhail Makarkin, Dmitry Gorin, Daniil Bratashov

**Affiliations:** 1 Science Medical Center, Saratov State University, 83 Astrakhanskaya Str., 410012 Saratov, Russia; r.a.verhovskiy@sgu.ru (R.V.); ermakov_a_v_2@staff.sechenov.ru (A.E.); prikhozhdenkoes@sgu.ru (E.P.); anastasiia.kozlova245@gmail.com (A.K.); grishin.optics@gmail.com (O.G.); makmih50@gmail.com (M.M.); 2 Department of Biomedical Engineering, Institute for Molecular Medicine, I. M. Sechenov First Moscow State Medical University, 8 Trubetskaya Str., 119991 Moscow, Russia; 3 Skolkovo Institute of Science and Technology, 3 Nobelya Str., 121205 Moscow, Russia; O.Sindeeva@skoltech.ru (O.S.); D.Gorin@skoltech.ru (D.G.)

**Keywords:** polyelectrolyte microcapsules, biodistribution, circulation time, immune system, magnetic delivery

## Abstract

Drug carriers based on polyelectrolyte microcapsules remotely controlled with an external magnetic field are a promising drug delivery system. However, the influence of capsule parameters on microcapsules’ behavior in vivo is still ambiguous and requires additional study. Here, we discuss how the processes occurring in the blood flow influence the circulation time of magnetic polyelectrolyte microcapsules in mouse blood after injection into the blood circulatory system and their interaction with different blood components, such as WBCs and RBCs. The investigation of microcapsules ranging in diameter 1–5.5 μm allowed us to reveal the dynamics of their filtration by vital organs, cytotoxicity, and hemotoxicity, which is dependent on their size, alongside the efficiency of their interaction with the magnetic field. Our results show that small capsules have a long circulation time and do not affect blood cells. In contrast, the injection of large 5.5 μm microcapsules leads to fast filtration from the blood flow, induces the inhibition of macrophage cell line proliferation after 48 h, and causes an increase in hemolysis, depending on the carrier concentration. The obtained results reveal the possible directions of fine-tuning microcapsule parameters, maximizing capsule payload without the side effects for the blood flow or the blood cells.

## 1. Introduction

Modern systems for targeted drug delivery solve several pharmaceutical problems, protecting the substance from biological media, allowing for the delivery of poorly soluble substances, and ensuring a better distribution of the substance throughout the body and over time. The main advantage of using drug delivery systems is the reduction in the systemic side effects of drug administration through local delivery to the affected area [1]. However, this approach becomes more complicated in the case of drug delivery to soft tissues and the internal parts of the body. In this case, the required drug distribution can be provided by the introduction of carriers into the bloodstream and localization in the affected area by external stimuli. One of the most promising approaches for control over the microcarriers is the magnetic field due to its high penetration level into biological tissues [2]. However, the high speed of the bloodstream [3] significantly complicates the accumulation of carriers’ in the affected area [4]. To be attracted and held against the vessel wall, the carriers must be sufficiently sensitive to the magnetic field. This requires the inclusion of nanoparticles with strong magnetic properties inside the carrier’s structure, which may lead to various side effects caused by the nanoparticles themselves, and a change in the properties of the carrier [5].

The main parameters, in terms of the safety and efficiency of drug delivery systems, are their load capacity, biocompatibility, biodegradability [6], sensitivity to external stimuli [4], duration of their circulation in the vascular system [7], and their ability to avoid the host’s immune system stimulation. Although the above parameters are crucial for successful targeted drug delivery, the carrier size can also play an important role. The increase in the carrier’s diameter allows for an increase in the amount of magnetic material inside the carrier, enhancing its sensitivity to the external magnetic field. Additionally, the enlargement of the drug delivery system carriers provides a higher loading capacity for pharmacologically active substances and magnetic nanoparticles for remote control. Moreover, the magnetophoretic velocity of a carrier significantly increases with an increase in diameter [8]. On the other hand, a larger carrier size results in faster filtration from the bloodstream, decreasing the circulation time [9] and altering the biodistribution of the delivered carriers and substances. Thus, the success of carrier targeting and therapeutic strategy for a specific disease will directly depend on the strength and duration of the magnetic exposure, carrier size, circulation time of a specific carrier type, and their biodistribution pattern. Moreover, impaired blood flow in the capillary system can lead to a decrease in the blood supply to tissues and organs [7,10,11]. Thus, all the aforementioned properties of remotely controlled drug delivery systems must be properly investigated prior to its clinical use.

The widely used layer-by-layer (LbL) assembly method offers excellent opportunities to synthesize microcarriers based on biocompatible organic polymers and inorganic functional fillers, including magnetic nanoparticles (MNPs) [4,12]. Polymeric capsules intended for systemic administration, such as blood cells, can significantly deform when passing through a small capillary due to their soft shell and hollow structure [7]. This makes them more beneficial compared with solid carriers. The possibility of using low-foaling polymers, which do not provoke immune recognition, as a material for assembling the carrier also indicates the promising use of polymeric capsules for systemic administration [13].

One of the most commonly used combinations of biocompatible and biodegradable polymers for LbL assembly is polyarginine (Parg)/dextran sulfate (DS), indicated by the tremendous number of articles published in the last decade [6,7,10,11,14,15,16]. However, despite the profound interest in magnetic Parg/DS microcapsules, the cytotoxicity, hemotoxicity, and systemic side effects induced by their application have not been properly studied to date. Previous studies have shown the biodistribution of magnetic Parg/DS microcapsules in the presence/absence of an external magnetic field and duration of their circulation in the blood-vascular system for systemic administration [7,10,11]. The biocompatibility of Parg/DS microcapsules has been proven in previous studies [5,17], while the cytotoxicity and hemotoxicity of magnetic microcapsules have not been properly investigated.

The efficiency of the magnetic trapping of microcapsules in the flow conditions, the duration of their circulation in the vascular system, and their biodistribution, depending on the capsules’ size and Fe_3_O_4_ NPs’ concentration, were evaluated in this study. These parameters are crucial for the application of magnetic microcapsules for remotely controlled drug delivery with systemic administration. In addition, the cytotoxic effect of magnetic microcapsules on murine RBCs (hemotoxicity) and macrophages (Raw 264.7 cell line), depending on capsule size, was evaluated, as well as the efficiency of their internalization by Raw 264.7 cells after 3, 6, 12, and 24 h of co-incubation. This allows for a comprehensive assessment of the effect of magnetic microcapsules on most common blood components.

## 2. Materials and Methods

### 2.1. Materials

Iron (III) chloride hexahydrate (99.8%), iron (II) chloride tetrahydrate (99.8%), sodium hydroxide (99.8%), citric acid (99.8%), sodium carbonate, calcium chloride, sodium chloride, poly-L-arginine hydrochloride (Parg, Mw = 15–70 kDa), dextran sulfate sodium salt (DS, Mw = 100 kDa), bovine serum albumin (BSA, lyophilized powder), ethylenediamine tetraacetic acid disodium salt (EDTA), phosphate-buffered saline (PBS, 0.01 M), ammonium rhodanide (NH_4_SCN), and Dulbecco’s modified Eagle medium (DMEM) with high glucose content were obtained from Sigma-Aldrich (St. Louis, MO, USA). Glycerin was purchased from Reachem (Moscow, Russia). Fetal bovine serum (FBS), penicillin–streptomycin solution, 0.25% trypsin solution with 0.02% EDTA, DAPI Solution (1 mg/mL), Alexa Fluor 488 Phalloidin, and the 0.4% Trypan blue solution were obtained from Thermo Fisher Scientific (Waltham, MA, USA). All chemicals were used without additional purification. Deionized water (specific resistivity higher than 18.2 MΩ·cm) from Milli-Q Direct 8 (Millipore, Merck KGaA, Darmstadt, Germany) water purification system was used to prepare all solutions.

### 2.2. Magnetite NPs Synthesis

The method of magnetite NPs synthesis was described previously in [18]. In brief the synthesis implies the chemical precipitation of iron (II) and iron (III) salts in the alkaline medium in inert atmosphere at 40 °C, followed by stabilization in 0.019 M citric acid. Then, the suspension of stabilized magnetite NPs was dialyzed for 3 days in 3 L of deionized water under slow mixing. After dialysis, the magnetite NPs concentration was evaluated by by colorimetric titration, based on the qualitative reaction of Fe^3+^ ions with ammonium thiocyanate [18], and was found to be 1 mg/mL. According to TEM images, the particles have a spherical shape with an average diameter of 5.4 ± 0.9 nm. The average hydrodynamic size of the stabilized magnetite NPs evaluated with DLS was around 8.3 ± 2.4 nm.

### 2.3. Preparation of FITC-Conjugated BSA

First, 160 mg of BSA were dissolved in 40 mL of 0.1 M PBS buffer (pH 8). Then, the solution of FITC in ethanol (5 mg/mL) was prepared. 40 mL of BSA solution was added to 5 mL of FITC alcoholic solution under gentle stirring and further the mixture was stirred under 4 °C in the dark for 12 h. Finally, freshly prepared FITC-conjugated BSA was dialyzed for 3 days in deionized water.

### 2.4. Preparation of Different Size Magnetic Fluorescent Microcapsules

The capsules were prepared following the traditional layer-by-layer (LbL) technique using colloidal microparticles as a template. The CaCO_3_ microparticles of different sizes were synthesized by mixing CaCl_2_ and Na_2_CO_3_. Protocols of CaCO_3_ synthesis were slightly changed to obtain the desired sizes. In brief, microparticles of larger sizes (2.7 and 5.5 μm) were obtained using a water subphase and a slower agitation rate (700 rpm for the synthesis of submicron microparticles, 500 rpm for 2.7 μm particles, and further reduced to 100 rpm to synthesize 5.5 μm particles) in the presence of highly concentrated salt solutions (1 M). In contrast, 1 μm microparticles were synthesized in the viscous environment (ethylene glycol) using 0.1 M solutions of the salts. The obtained CaCO_3_ spherical particles were collected by centrifugation and thoroughly washed with deionized water. Multilayer capsules comprising 4 bi-layers of Parg/DS were then assembled on the surface of CaCO_3_ via the LbL technique. DS and Parg were alternatively adsorbed from 2 mg/mL (for 1 μm capsules due to increased surface area) and 1 mg/mL (for 2.7 and 5.5 μm capsules) aqueous solutions, also containing 0.5 M NaCl, starting from the Parg layer. The obtained coated particles were treated with 5 mL of 0.2 M EDTA for 15 min to remove CaCO_3_ cores, resulting in hollow polymeric capsules.

To load microcapsules with magnetite nanoparticles, prior to shell formation, the CaCO_3_ particles (40 mg per sample) were resuspended in 1 mL of magnetite nanoparticle colloid and frozen under continuous agitation according to the previously reported method [19]. The number of freezing/thawing cycles was ranged from 1 to 3 times to increase the number of magnetite nanoparticles immobilized within CaCO_3_ cores. Subsequent dissolution of the cores with EDTA after the polyelectrolyte shell formation resulted in the release and re-adsorption of the nanoparticles onto the internal surface of the polyelectrolyte shells. In another case, magnetite nanoparticles were assembled as the first layer of the polyelectrolyte shells during the LbL procedure as a negatively charged species. The concentration was chosen depending on the microcapsules’ surface area to maintain the average distance between nanoparticles in the shell—1 mg/mL of magnetite nanoparticles for 1 μm CaCO_3_, 0.371 mg/mL for 2.7 μm and 0.186 mg/mL for 5.5 μm particles.

### 2.5. Characterization of Magnetite Nanoparticles and Microcapsules

The morphology and size of magnetite nanoparticles and polyelectrolyte microcapsules were studied by transmission (TEM) and scanning (SEM) electron microscopy. TEM imaging was performed with a FEGTEM microscope (JEOL, Akishima, Tokyo, Japan) operating at 200 kV. Samples for TEM were prepared by drying a drop of the aqueous suspension of nanoparticles on the lacey-carbon copper grid. SEM was performed with MIRA II LMU (Tescan, Brno, Czech Republic) microscope at an operating voltage of 30 kV in secondary and backscattering electron modes. Brightfield and fluorescence images of microcapsules and cells were made using ImageStream X Mark II Imaging Flow Cytometer (Merck, Kenilworth, NJ, USA).

### 2.6. Device Design

**Figure 1:**
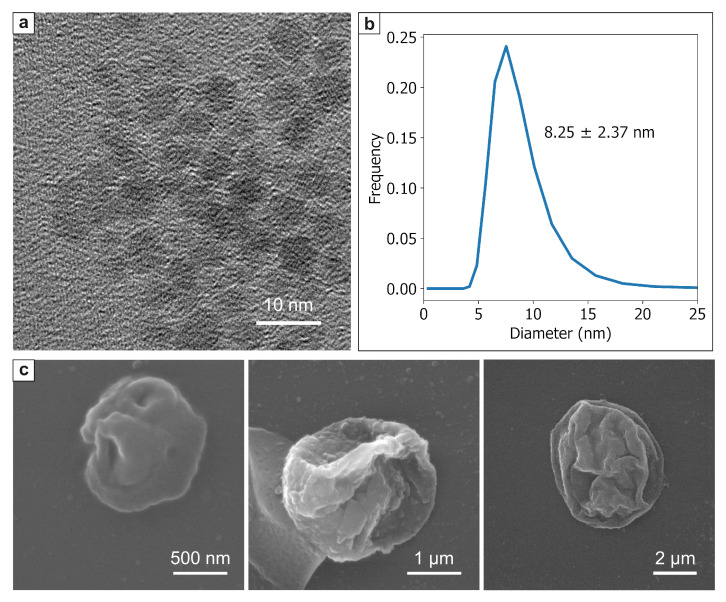
Shape and size of MNPs and polyelectrolyte microcapsules: (**a**) TEM image and (**b**) DLS size distribution of Fe_3_O_4_ NPs; (**c**) SEM images of 1 (left panel), 2.7 (middle panel), 5.5 μm (right panel) Parg/DS microcapsules.

The optical design of the device is based on a simplified variant of the SPIM-Fluid imaging flow cytometer [20] and described in detail in our previous publication [14]. The major features are shown in Figure 1. We are making a simplified lightsheet microscopy system where the light sheet is formed by a pair of 50 mm cylindrical lens and illumination objective lens 4×/NA 0.13 Nikon objective. We used 488 nm laser Cobolt 06-MLD (HÜBNER Photonics, Kassel, Germany) with a 3.5× beam expander as illumination source. The detection system in this variant is based on 10×/NA 0.3 Nikon objective lens, a set of fluorescent filters, where MF530-43 (Thorlabs, Newton, NJ, USA) FITC line filter was used in our experiments, a tube lens with f = 200 mm and sCMOS camera Dhyana BSI400 (Tucsen, Gaishan Town, China). In the flow system, a flow-through quartz UV cell (526UV0.25, FireflySci, Northport, NY, USA) with a 0.3 T rare-earth magnet and a conical-shaped magnetic field concentrator was used. An AL-1000 syringe pump (World Precision Instruments, Sarasota, FL, USA) was used to provide the flow in the flow cell at the required rate.

### 2.7. In Vitro Experiments

#### 2.7.1. The Efficiency of Microcapsules Capture by the Magnetic Field

The efficiency of the microcapsules’ capture by the external source of the magnetic field was evaluated using the custom-built, lightsheet-based flow cytometer [14]. For this, the suspension of capsules labeled with FITC was passed through the flow cell with a velocity of around 30 μL/min. During the capsules’ passing, 0.3 T permanent magnet moved up to the flow cell, and magnetic capsules accumulated on its internal wall. The estimation of captured capsules number was performed in three ways: by the analysis of average fluorescence intensity within the region of interest (ROI), counting the objects using the computer vision method, and by the enumeration of captured capsules using imaging flow cytometry system Image Stream X Mark II.

##### Analysis of Average Fluorescence Intensity

This method is based on counting the average intensity of fluorescence within the ROI that corresponds to the lightsheet plane image on camera. The integral fluorescent signal inside the ROI is somewhat proportional to the amount of fluorescent material within the lightsheet plane and the number of capsules in the lightsheet plane can be estimated from it. Despite its simplicity, if we have a single type of uniform fluorescent objects in the flow with no background, it provides a rather good estimation of flowing capsules number if no intensity clipping by the dynamic range of the camera occurs.

##### Computer Vision Method

The number of magnetic capsules in the light sheet plane was used a metric for this counting method. The computer vision method involves counting the number of capsules by selecting and counting the closed contours of fluorescently marked objects in the case of single capsules and analyzing the average fluorescent area in the case of large clusters of capsules. Adjacent frames are subtracted to remove static objects. Before applying a magnetic field to the flow cell, small fluorescent objects flowing through the light-sheet plane were usually observed, which corresponded to the single carriers. Each contour corresponded to an independent magnetic capsule. However, the application of a magnetic field results in bright aggregates of capsules on the internal wall of the flow cell, making it impossible to count the single capsules inside. For this reason, the average area of a single capsule before the magnet application was evaluated, and then these data were used to estimate a capsules’ number in the aggregate by its projected area on each image. The beginning of the formation of particle aggregates corresponds to the timepoint when the number of contours drops sharply, along with their rapid increase in area. Then, we divide the area of the aggregate contour by the average area of the individual capsule’s contour. Since the size of the aggregates is considered proportional to the number of capsules that stick together, the resulting number will be a rough estimate of the total number of microcapsules in the aggregate.

All programs were written in Python using an open source library OpenCV.

##### Analysis by Imaging Flow Cytometry System

The capsules that accumulated on the internal wall of the flow cell were counted using ImageStream X Mark II Imaging Flow Cytometer. For this, the magnet was removed and the accumulated capsules were washed out by clean PBS and collected into 1.5 mL tube. Then, capsules were resuspended in PBS and measured at low-flow-rate/high-sensitivity mode using INSPIRE software. The capsules’ fluorescence was excited by the 488 nm laser operating at 100 mW power. Flow cytometry data were processed using IDEAS software, as was described previously [7,16].

#### 2.7.2. Cell Preparation

The murine macrophages cell line (Raw 264.7) was provided by the Science Medical Center, Saratov State University, Russia. Cells were grown to confluence in Dulbecco’s modified Eagle’s medium containing 4500 mg/L of glucose supplemented with 10% of FBS, and 1% of penicillin-streptomycin. Cells were cultivated in a humidified atmosphere of 5% CO_2_ at 37 °C. The medium was replaced every 3 days. After the monolayer formation, cells were detached using 0.05% trypsin with EDTA and counted by the Countess automated cell counter (Thermo Fisher Scientific, Waltham, MA, USA).

#### 2.7.3. Cell Viability

The cytotoxic effect of different size magnetic polyelectrolyte microcapsules on the Raw 264.7 cell line was evaluated using AlamarBlue cell viability reagent. In brief, cells were seeded into 96-well plates at 10${}^{4}$ cells per well and cultivated overnight. Then, different size capsules were added to cells in 1, 5, 10, and 50 particles per cell concentration, and co-incubated for 24 and 48 h. Cells incubated without capsules were used as a positive control. After that, 10 μL of AlamarBlue reagent was added to each well with 100 μL of cultural medium and incubated for 4 h. In the last step, fluorescent (560/590 nm) intensity was measured by the multimode microplate reader Synergy H1 (BioTek, VT, USA). All obtained fluorescence intensity data were normalized on the control sample [21].

#### 2.7.4. RBCs Hemolysis

The level of RBCs hemolysis after 24 h co-incubation with different-size magnetic polyelectrolyte microcapsules was evaluated using murine whole-blood. For this, blood was centrifuged at 400× *g* for 10 min, cells’ pellet was triple-washed by DPBS and then re-suspended in DPBS at a concentration of 10% red blood cells by volume [22]. Suspension of RBCs was divided into three parts: negative control (cells lysed with pure water), sample (RBCs co-incubated with microcapsules for 24 h), and control (RBCs incubated for 24 h). All data were normalized on negative control. For the hemolysis evaluation, RBC suspension was centrifuged and the supernatant was collected for further analysis. The RBCs’ hemolysis level was estimated by measuring free hemoglobin absorption in the DPBS at 540 nm [22] by Synergy H1 microplate reader. All obtained optical density (OD) values were normalized on DPBS OD.

#### 2.7.5. Capsules’ Internalization Efficiency

The efficiency of internalization of 1, 2.7, and 5.5 μm magnetic polyelectrolyte microcapsules labeled with TRITC was investigated on the Raw 264.7 cell line. Cells were cultured in the presence of carriers in the concentration of 10 capsules per cell for 3, 6, 12, and 24 h. The visualization of cells that internalized polyelectrolyte microcapsules was performed using an Amnis ImageStream X Mk II imaging flow cytometer. For this, cells were triple-washed by PBS to remove uncaptured capsules and detached from the Petri dishes using trypsin-EDTA solution. Then, cells were fixed by 10% neutral buffered formalin at 4 °C for 10 min, washed with PBS and stained with 1 μg/mL of Alexa Fluor 488 phalloidin and 1 μg/mL of DAPI for 30 min. Finally, cells were resuspended in the appropriate volume of PBS and measured by imaging flow cytometry. The data were analyzed using IDEAS 6.2 software (Luminex). The number of containers captured by a cell was evaluated with the "Internalization" and "Spot counter" software tools designed to detect and enumerate intracellular fluorescent-labeled objects.

### 2.8. In Vivo and Ex Vivo Experiments

Experiments were performed on Balb mice 6–8 weeks old with an average weight of 20–25 g under general anesthesia (Zoletil mixture, 50 μL, 40 mg/kg (Virbac SA, Carros, France) and 2% Rometar, 10 μL, 10 mg/kg (Spofa, Czech Republic) via intraperitoneal injection). All experimental procedures were carried out according to the rules of Saratov State Medical University (Ethics Committee protocol no. 5, 29 December 2018). A microcapsule suspension (0.2 mL; 1, 2.7, and 5.5 μm in size) was injected into the tail vein at a dosage of 10${}^{7}$ microcapsules per mouse. After the experiment was completed, the animals were sacrificed using an overdose of anesthesia.

#### 2.8.1. Estimation of Microcapsules Circulation Time

Then blood sampling was performed from retro-orbital sinus upon 0.5, 1, 3, 5, and 15 min after capsule injection. After that, blood samples (10 μL) were diluted 200-fold by PBS buffer to obtain the optimal cell concentration. Then, equal sample volumes (10 μL) were measured for each timepoint by ImageStream X Mark II Imaging Flow Cytometer. Measurements were performed using INSPIRE software at 40× magnification and low-flow-rate/high -ensitivity mode, as described previously [7]. The sample fluorescence was excited by the 488 nm laser with 100 mW power.

#### 2.8.2. Estimation of Microcapsule Biodistribution

The microcapsule biodistribution in vivo at 15 min after tail vein injection was imaged using the IVIS SpectrumCT In Vivo Imaging System (PerkinElmer, Waltham, MA, USA) using excitation/emission at 745/800 nm. An analysis of the results was carried out using Living Image software 4.7.3. The organs of experimental animals were imaged ex vivo, measured and normalized by the intact mice organs (without any injection). Total Radiant Efficiency values were obtained for each region of interest. ROIs were selected to match the boundaries of the organs. Total radiant efficiency is an integrated photon amount per second from the selected area, normalized to illumination intensity.

## 3. Results

This study analyzed the behavior of remotely controlled drug delivery systems based on Parg/DS microcapsules under systemic administration to mice. In the course of the study, the size of the microcapsules and the number of magnetite nanoparticles introduced to the shell were varied. The number of magnetite particles was varied using the method of introduction, either label-by-label (LbL) or freezing-induced loading (FIL) [19]. Since FIL can be applied repeatedly, by increasing the amount of substance introduced into the shell, the number of magnetite nanoparticle loads using this approach was varied from 1 to 3.

### 3.1. Characterization of Microcarriers

According to transmission electron microscopy (TEM) analysis, the particles were characterized by a spherical shape and an average size of 5.4 ± 0.9 nm (Figure 1a). The average hydrodynamic radius of the stabilized MNPs measured by dynamic light scattering (DLS) was found to be about 8.25 ± 2.37 nm Figure 1b). Microcapsules were prepared by sequential LbL assembly of Parg and DS polyelectrolytes on the surface of colloidal calcium carbonate (CaCO_3_) microparticles with three different sizes: 1, 2.7, and 5.5 μm, which correspond to the sizes of the resulting microcapsules Figure 1c).

The parameters of magnetic nanoparticles indicate that they should have good magnetic properties if they are not oxidized, as provided by an inert medium during synthesis. The layer of the stabilizer separating them has sufficient thickness to prevent the strong interaction between individual particles; therefore, the polyelectrolyte shell can be considered a polymer composite with separated individual magnetite particles. SEM images show a characteristic flattened composite shell bilayer of noticeable thickness, most of which is formed by magnetite nanoparticles. Upon drying, no tears in the shell were observed, showing that it has sufficient mechanical strength.

### 3.2. Evaluation of Microcapsules’ Capturing by the Magnetic Field

**Figure 2:**
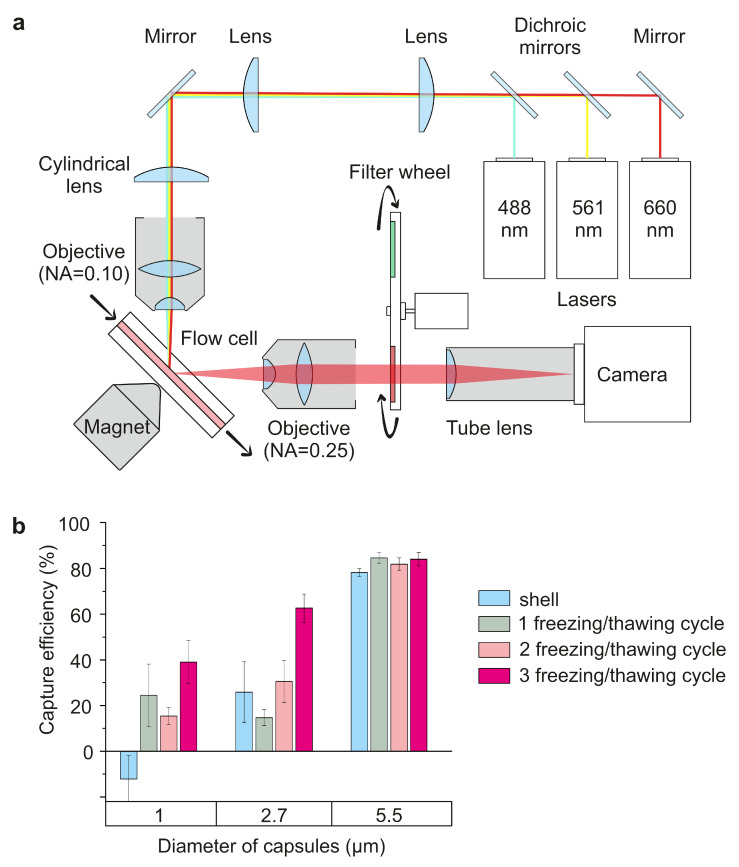
Relationship between carrier size and loaded MNPs’ concentration and efficiency of microcapsule capture: (**a**) experimental setup schematics; (**b**) capture efficiency of 1, 2.7, and 5.5 μm magnetic-sensitive microcapsule from the flow with 30 μm/min flow velocity.

First, the efficiency of magnetic-sensitive microcapsule capture by the external source of the magnetic field was studied in vitro in terms of dependence on carriers’ size and MNPs concentration. For this, Parg/DS microcapsules were fluorescently labeled by adding the fluorescein-conjugated bovine serum albumin (BSA-FITC) molecules in the structure of the capsules shell. The shell structure was designed so that the magnetite layer was separated from the BSA-FITC layer by several layers of polyelectrolytes to avoid fluorescence quenching by the nanoparticles. Thus, four different configurations of 1, 2.7, and 5.5 μm microcapsules were synthesized. The structure of LbL-assembled capsule shell was (MNPs)${}_{n}$/(Parg/DS)${}_{2}$/Parg/BSA-FITC/Parg/DS, where *n* is number of FIL cycles. Then, different types of microcapsules suspended in phosphate saline buffer (PBS) were passed through the flow cell of the previously described, light-sheet-based flow cytometry system [14]. The flow velocity was around 30 μL/min (≈10 mm/s), which is close to the blood flow velocity in femoral veins of small laboratory animals [14]. The magnetic capture of synthesized microcapsules was performed using the 0.3 T permanent magnet equipped with the conical concentrator, the apex of which was placed close to the flow cell’s wall, as presented in Figure 2a.

Capsule capture efficiency was assessed in three ways: by analyzing the average fluorescence intensity in the region of interest (ROI) (see Supplementary Materials, Figure S1, blue graph) [14], estimating the capsule number using computer vision techniques (Supplementary Materials, Figure S1, red graph), and counting the number of captured capsules using an imaging flow cytometry system (Figure 2b). The increase in the efficiency of capsule capture by the external magnetic field with increases in carrier size and MNP concentration in the capsule has been demonstrated. Therefore, carriers with MNPs in the shell demonstrated the lowest sensitivity to the magnetic field. The difference in the magnetic field sensitivity of 1 μm capsules with 1 and 2 freezing/thawing cycles was statistically insignificant, while capsules with 3 freezing/thawing cycles were more than 1.5 times more sensitive to the magnetic field. The same tendency was observed for 2.7 μm capsules, but 5.5 μm capsules’ sensitivity to the magnetic field was nearly the same, independent of the number of FIL cycles, presumably due to their higher load capacity. A large capsule surface area and high payload ability of 5.5 μm vaterite particles allow for a sufficient amount of MNPs to be loaded into carriers, making them sensitive enough to the magnetic field even upon the inclusion of MNPs in the shell or at one freezing/thawing cycle. Nonetheless, capsules with three freezing/thawing cycles showed the best magnetic field sensitivity for each size, so this carrier type was used in all the following experiments.

### 3.3. Evaluation of Microcapsules’ Circulation Duration and Their Biodistribution

**Figure 3:**
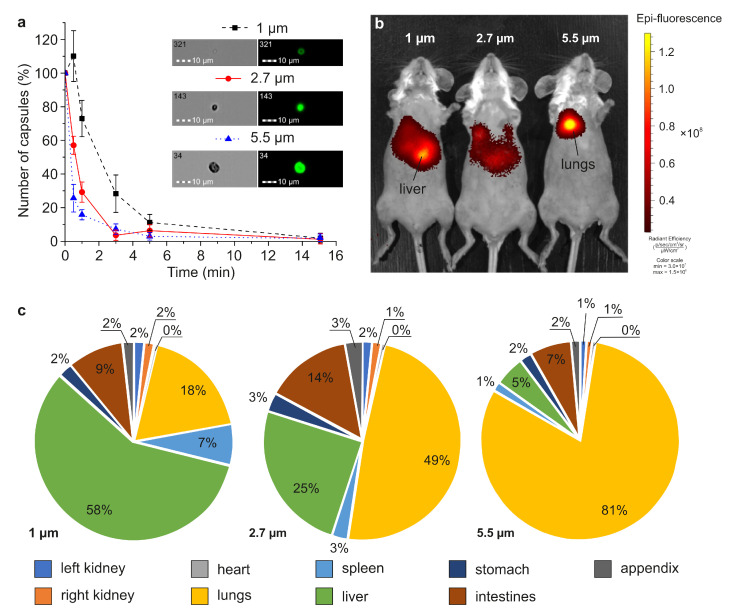
The circulation duration and biodistribution of 1, 2.7, and 5.5 μm magnetic capsules: (**a**) The number of circulating capsules after 0.5, 1, 3, 5, and 15 min after the injection, normalized on the number of injected capsules; (**b**,**c**) the magnetic capsules biodistribution upon 15 min after the injection (**b**) in vivo and (**c**) ex vivo.

The duration of circulation for capsules of different sizes, and their biodistribution in the body of mice after injection into the tail vein, were studied. Capsules were labeled with FITC ((MNPs)${}_{3}$/(Parg/DS)${}_{2}$/Parg/BSA-FITC/Parg/DS for the investigation of circulation duration) and Cy7 ((MNPs)${}_{3}$/(Parg/DS)${}_{2}$/Parg/BSA-Cy7/Parg/DS for the investigation of biodistribution). Capsules in saline (10${}^{7}$ capsules per 200 μL) were injected in the tail vein, and blood was sampled from retro-orbital sinus upon 0.5, 1, 3, 5, and 15 min after injection. Then, the obtained sample was diluted 200-fold with PBS, and the number of capsules was estimated with the Imaging flow cytometry system (Figure 3a). A decreasing tendency in the number of circulated capsules related to the increase in their size was revealed. The difference in the percentage of circulating capsules for all sizes was most significant in the first 30 seconds, and still significant in the first minute: 72, 30, and 18% for 1, 2.7 and 5.5 μm respectively. In the third minute after the injection, only 30% of 1 μm capsules were still circulating in the blood flow, while different organs had already filtered more than 90% of 2.7 and 5.5 μm carriers. More than 95% of the capsules had been filtered by organs by 15th minute, regardless of their size. Therefore, an investigation of their biodistribution at this timepoint seems reasonable, and is described below.

The biodistribution of fluorescently labeled magnetic microcapsules was studied ex vivo and in vivo (Figure 3b,c). The capsules of 1 μm in size preferentially accumulated in the liver, and 5.5-μm-sized capsules were predominantly accumulated in the lungs. In the case of intravenous administration of 1 μm capsules, the proportion of the fluorescent signal from the liver and lungs after 15 min was 58% and 18%, for 2.7 μm 25% and 49%, and 5.5 μm 5% and 81%, respectively.

### 3.4. Evaluation of Microcapsules’ Effect on the Blood Cells

**Figure 4:**
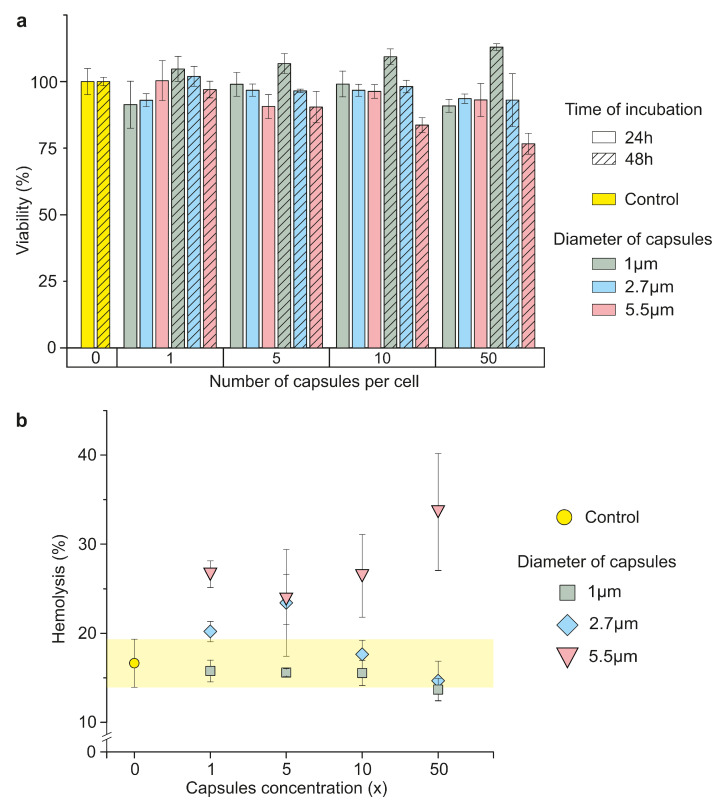
Impact of magnetic microcapsules of different sizes (1, 2.7 and 5.5 μm) on mouse blood cells: (**a**) Raw 264.7 cell viability after 24 and 48 h incubation with microcapsules; (**b**) the degree of lysis of mouse erythrocytes after 24 h of incubation with microcapsules (control hemolysis values are shown with yellow).

The effect of magnetic Parg/DS microcapsules on different components of murine blood depending on the capsules’ size was studied in vitro. The viability of the murine macrophage cell line (Raw 264.7) was estimated after 24 and 48 h of incubation in presence of Parg/DS microcapsules in the medium at concentrations of 1, 5, 10, and 50 capsules per cell. Cells without capsules in the medium were considered a positive control [21]. The minimal capsules concentration In vitro corresponds to the concentration used for the in vivo experiments. Microcapsules with average sizes 1 and 2.7 μm did not significantly influence cell viability in all concentrations after 24 h and 48 h of incubation (Figure 4a), which correlates with the data presented in [6]. The concentration-dependent cytotoxic effect was found in the case of 5.5 μm capsules for 10 and 50 capsules per cell after 48 h of incubation. 5.5 μm carriers in the concentration of 50 particles per cell lead to more than a 25% decrease in cell viability after 48 h of incubation compared to the control.

Since the number of RBCs in the whole blood is essentially higher than WBCs, in the assessment of hemolysis, equivalent capsule concentrations (1×, 5×, 10×, and 50×) to those applied in previous in vitro and in vivo experiments were used. For hemolysis evaluation, RBCs re-suspended in DPBS at a concentration of 10% by volume [22] was divided into three groups: negative control (cells lysed with pure water), sample incubated with capsules, and the non-treated sample. RBCs’ hemolysis was evaluated by measuring the absorption of hemoglobin leaked from damaged RBCs into DPBS at 540 nm [22]. The values obtained for the solutions’ optical density (OD) were normalized on DPBS OD.

The RBCs’ hemolysis values after 24 h of incubation with all studied concentrations of 1 and 2.7 μm magnetic capsules lie in the control range (Figure 4b, yellow region). The incubation of murine RBCs with 5.5 μm capsules for 24 h led to an increase in hemolysis depending on capsule concentration, which correlates with the data obtained for the macrophage cell line (Figure 4b, red triangles). Therefore, all concentrations of 1 and 2.7 μm and a low concentration (1 and 5 capsules per cell) of 5.5 μm magnetic Parg/DS capsules do not affect murine blood cell, while 10 and 50 capsules per cell concentration of 5.5 μm carriers cause a decrease in macrophage’s cell viability and an increase in hemolysis.

### 3.5. Evaluation of Microcapsules’ Internalization Efficiency by Macrophages

The efficiency of internalization of magnetic microcapsules of different sizes with the Raw 264.7 cell line was also estimated. For this, capsules fluorescently labeled with TRITC (MNPs)${}_{3}$(Parg/DS)${}_{2}$/Parg/BSA-TRITC/Parg/DS) were added to the attached cells at a concentration of 10 particles per cell, followed by incubation for 3, 6, 12, and 24 h. Then, cells were detached, fixed with 4% paraformaldehyde in PBS, and fluorescently stained with DAPI (cell nucleus) and Alexa Fluor 488 phalloidin (cytoskeleton). An analysis was performed using the Image Stream®X Mark II imaging flow cytometry system.

**Figure 5:**
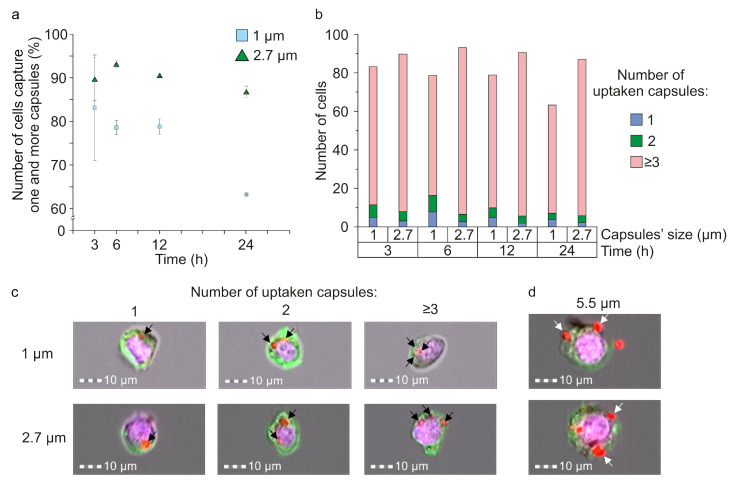
The efficiency of 1, 2.7, and 5.5 μm microcapsules internalization by Raw 264.7 cells: (**a**) The number of cells that captured one or several capsules with an average diameter of 1 and 2.7 μm, depending on time; (**b**) distribution of the number of cells that internalized 1, 2, 3 or more capsules after 3, 6, 12 and 24 h of simultaneous incubation; (**c**) brightfield/fluorescent images of Raw 264.7 cells that captured 1, 2, and 3 and more of 1 and 2.7 μm capsules (black arrows indicate internalized capsules); (**d**) brightfield/fluorescent images of Raw 264.7 cells after interaction with 5.5 μm capsules (white arrows indicate capsules sorbed on the cells’ surface).

It was found that the most intense capturing of magnetic microcapsules by macro-phage occurred in the first 6 h (Figure 5a). The comparison of the internalization efficiency of 1 (blue square) and 2.7 μm (green triangles) capsules showed that macrophages most effectively capture 2.7 μm capsules at all timepoints (Figure 5a). Therefore, after 6 h of incubation, 93.1 ± 0.8% of cells captured one and more of 2.7 μm capsules, while for 1 μm carriers, this value was 78.6 ± 1.6%. Further incubation for 12 and 24 h demonstrated a decrease in the number of cells that capture microcapsules caused by cell proliferation. A detailed analysis of the number of cells that captured 1, 2, and 3 and more capsules showed the decreasing tendency for cells that internalized 1 and 2 capsules, while the number of cells with 3 and more capsules increased over time (Figure 5b,c).

Analysis of the internalization of 5.5 μm capsules was complicated due to their large size. The capsule volume in this case was one-sixth of the average cell volume. Capsules and cells lying in different focal planes or capsules in contact with the cell surface were usually found (Figure 5d, white arrows), which indicates a low efficiency of their internalization. Thus, it was difficult to tell whether such large objects were internalized by cells or simply attached to the cell wall.

## 4. Discussion

Magnetic polyelectrolyte microcapsules are promising carriers, opening the possibilty of targeted, controlled drug delivery into the affected area. The magnetic properties of these carriers are usually provided by the magnetite nanoparticles (Fe_3_O_4_) included in their structure. Although iron oxides are presented in the human body [23], high concentrations of MNPs were found to be toxic [24]. Application of the magnetic capsules suggests a systemic administration by injecting the microcapsule suspension into the blood flow, which is followed by targeting the affected area using an external source of magnetic field [4,25]. In this regard, the study of microcapsule behavior in the blood seems relevant when testing the drug delivery system.

The most important characteristic of targeted magnetic carriers is the efficiency of their capture by the magnetic field. This parameter was evaluated by an analysis of the data obtained using a custom-built, lightsheet-based flow cytometer and commercial imaging flow cytometry system. The data from the custom-built system were analyzed by the enumeration of the capsules’ number in the frame (computer vision method) or by a comparison of average fluorescence intensity within the ROI before and after the application of the magnetic field to the flow cell [14]. The data obtained by the computer vision method (see Supplementary Materials, Figure S1, red plot) and by the analysis of ROI’s fluorescence intensity (Supplementary Materials, Figure S1, blue plot) highly correlate, which allows us to use both methods to evaluate capsules’ capture efficiency.

The efficiency of trapping microcapsules by a magnetic field substantially depends on their structure and size (Figure 2b), which is mainly explained by the difference in the hydrodynamic properties of the capsules and differences in the amount of magnetite in the structure of the capsules. Besides the composition of the shell, the microcapsule’s size should be carefully considered in these terms, as this affects circulation dynamics and biodistribution, and the process of intracellular internalization. The inclusion of Fe_3_O_4_ NPs into the shell of 1 and 2.7 μm capsules or their loading into the hollow cavity of capsules by a single freezing/thawing cycle both result in a low capsule sensitivity to the magnetic field. However, capsules of a larger size (5.5 μm) do not suggest that the sensitivity to the magnetic field is dependent on capsule configuration in terms of MNP localization. In addition, the largest capsule size exhibits a higher capture efficiency of up to 80%, although it is more susceptible to drifting downstream under the influence of flow. Therefore, an increase in the magnetite concentration, while keeping capsule size constant, leads to an enhancement in the magnetic sensitivity of carriers due to the linear increase in the previously shown loaded magnetite amount [19]. However, this tendency is observed up to a particular magnetite concentration, after which the saturation effect of the sensitivity on the magnetic field is observed. The obtained data have demonstrated that three freezing/thawing cycles provide the maximal sensitivity of carriers to the magnetic field crucial for biomedical application. However, it should be noted that an increased concentration of magnetite can induce cytotoxic effects [24] and systemic toxicity during microcapsules’ biodegradation within the body. It was previously shown [7] that the systemic introduction of polyelectrolyte microcapsules into the bloodstream of white rats does not cause irreversible disruptions in the blood supply to vital organs. However, a comparative investigation of carriers with different sizes has not been performed.

Here, we estimated the duration of circulation of 1, 2.7 and 5.5 μm capsules in the circulatory system of mice and determined a tendency for this time to decrease with an increase in the size of the carrier (Figure 3a). Fifteen minutes after the injection, the number of circulating capsules did not exceed 5% for all studied sizes that corresponds to the data obtained previously for 5-μm-sized magnetic [7] and 3-μm-sized non-magnetic capsules [11]. This is probably due to the rapid filtration of capsules in the small capillaries of the lungs (about 10 μm in diameter) [26] and livers’ sinusoids (about 10–15 μm in diameter) [27], which is confirmed by the capsules’ biodistribution data (Figure 3b,c). A decrease in the average carrier size led to a decrease in the number of capsules stuck in the lungs from 81% for 5.5 μm to 18% for 1 μm and an increase in the number of carriers filtered by the liver, from 5% to 58%, respectively (Figure 3c). It can generally be concluded that intravenous administration of 5.5 μm magnetic microcapsules is appropriate for targeting the lungs. Lungs are the first organs through which capsules pass after the injection, and most of them are passively accumulated in the lungs’ capillary system during the first passage. In this case, the magnetic exposure will most likely be directed not at accumulation, but at keeping the capsules stuck in the lungs. The magnetic targeting of 5.5-μm-sized capsules to other organs is likely to be the least effective (among the sizes studied). With a decrease in the capsules’ diameter to 2.7 and 1 μm, the time of their circulation in the bloodstream increases, which consequently increases the efficiency of their magnetic targeting to other organs. However, the optimal period of magnetic exposure for the accumulation of capsules will not exceed 3 and 5 min, respectively, since most of the capsules are filtered during this time period. The subsequent magnetic exposure will keep the accumulated capsules in the affected area. In vivo data show that magnetic targeting by intravenous delivery still has a limited effectiveness. Most of the dosage is accumulated in the lungs or liver [7] due to the systemic dispersion of capsules in vessels after the injection, which significantly limits the quantity of capsules passing through the affected area. The liver quickly filters foreign objects from the blood, since this is one of its main functions [28,29]. The injection of capsules through the artery supplying the affected area can significantly increase the effectiveness of the magnetic targeting [30] as it hits all capsules in this area for the first passage [31]. In this case, the 5.5 μm carriers are more appropriate for drug delivery, because they are mechanically stuck in the small capillaries supplying the affected region and can carry more cargo than the small ones. This can cause blood supply disruption and a shortage of oxygen supply to adjacent tissues. The ramifications of the organ’s capillaries network also affects the targeting effectiveness. We have previously shown that the capsules of a similar size accumulate in bifurcations and bends of the vascular bed [4]. However, the selection of the correct dosage is an extremely critical factor in the case of the arterial delivery, excluding the irreversible embolization of the vessels of the targeted organ [10,11].

Evaluation of the cytotoxicity of capsules with a size of 1 and 2.7 μm was carried out using the Raw 264.7 cell line, which showed no negative effects for up to 50 particles per cell during 24 and 48 h incubation of the cell culture (Figure 4a). This proves the high biocompatibility of this drug delivery system and corresponds to the data published elsewhere [6]. In addition, all investigated concentrations of 1 and 2.7 μm carriers do not cause additional hemolysis; after 24 h of incubation, it is at the level of the control sample (Figure 4b), which also proves the system’s safety. Moreover, the metabolic activity of the cells incubated with 1 μm capsules for 48 h was noted to be higher than that after 24 h at all studied concentrations, which shows the absence of the inhibition of the cell’s proliferation. 5.5 μm capsules are characterized by dose-dependent cyto- and hemotoxicity at concentrations of 10 and 50 capsules per cell, probably caused by the increased concentration of magnetite NPs. Therefore, applying 1 and 2.7 μm magnetic capsules using systemic administration is safer and does not induce damage to blood cells. However, the small carrier size significantly restricts their payload ability; this is why the balance between the loading efficiency and safety is a crucial characteristic of the developed drug delivery system.

Along with the circulation time, biodistribution, and cytotoxicity, the assessment of microcapsule internalization by the immune system cells is of great interest. The capture of magnetic microcapsules by macrophages mainly occurs in the first 6 h after their addition (Figure 5a). A longer incubation period seems to demonstrate the decrease in the number of the cells with internalized capsules caused by cells’ proliferation (Figure 5a). Furthermore, the detailed analysis revealed an increase over time in the percentage of cells, with more than three microcapsules captured. Surprisingly, 2.7 μm capsules exhibited a higher level of internalization at all timepoints, in comparison with 1 μm capsules. This effect could be explained by the lower probability of macrophages’ interaction with 1 μm capsules than 2.7 μm ones. In all experiments, cells’ confluency was around 60%; therefore, the distance between cells was around 20–25 μm. The enlargement of capsules’ size leads to the increased probability of cell interaction with the capsule and, consequently, its internalization. Theoretically, in the whole blood, we can observe a similar effect. Previously, we have shown the tendency for microcapsules to become isolated from each other by RBCs [16]. Since the number of RBCs in the whole blood is more than 1000 times higher than WBCs, the capsules are more likely to interact with RBCs than with immune cells. In contrast, 5.5 μm capsules can hardly be characterized in terms of internalization, since their size is comparable to the macrophage size, and microcapsules’ adsorption to the membrane occurs rather than internalization through the membrane (Figure 5c).

In this way, we can conclude that 1 μm capsules exhibit prolonged circulation periods accompanied by poor internalization by immune cells, which is beneficial for targeted drug delivery through systemic administration. However, 2.7 μm carriers demonstrate an improved internalization in comparison with 1 μm capsules, which is desirable for the intracellular delivery of drugs.

## 5. Conclusions

The characteristics of the carrier, such as size, shell composition, coating of the last layer, and loading of magnetic particles, define their utility parameters, such as sensitivity to a magnetic field, circulation time, biodistribution, cytotoxicity, and hemotoxicity and, thus, the efficiency of targeted drug delivery. A total of 1 and 2.7 μm capsules, heavily loaded with magnetite nanoparticles by three freezing/thawing cycles, were more sensitive to the magnetic field than configurations with a lower density of magnetite nanoparticles within the shell, independently of size. As a result, they can be more efficiently captured from the flow. 5.5 μm carriers were equally sensitive to the magnetic field independent of magnetite loading parameters due to the high load capacity of the large shell. After the systemic administration of polyelectrolyte microcapsules into the bloodstream, it has been demonstrated that the liver primarily filters 1 μm carriers, whereas an increase in capsule size led to their accumulation, predominantly in the lungs. Circulation time in the bloodstream decreases with an enlargement of the carriers’ average diameter. Most of the 5.5 μm capsules were filtered in the first 30 s during the first passage. However, 1 μm capsules demonstrated prolonged circulation in the mouse bloodstream, as they were filtered only after the first 3 min. An analysis of magnetic polyelectrolyte microcapsules’ effect on blood components has shown that 2.7 μm were more efficiently absorbed by macrophages, which indicates their effectiveness for intracellular delivery, while 1 and 5.5 μm capsules showed worse internalization characteristics. 5.5 μm capsules demonstrated the highest cyto- and hemotoxicity compared to 1 and 2.7 μm microcapsules. An enlargement of carrier size increases their payload ability; however, it leads to significant limitations regarding their biomedical application. The current work determines the limiting levels of microcapsule parameters that lead to adverse effects on microcapsules’ use for systemic administration. Based on these data, a finetuning of microcapsule parameters can be performed to increase drug payload while minimizing adverse effects and delivering the capsules’ to the targeted organ(s). This paves the way to more effective polyelectrolyte microcapsules’ use for the systemic administration of drugs through the bloodstream and effective magnetic accumulation, mostly in the affected region.

## Data Availability

Data underlying the results presented in this paper are not publicly available at this time but may be obtained from the authors upon reasonable request.

## Supplementary Materials

The following are available online at https://www.mdpi.com/article/10.3390/pharmaceutics13122147/s1, Figure S1: Dependence of the total fluorescence in the region of magnetic accumulation, and the calculated amount of polyelectrolyte microcapsules, on time after adding a magnet into the flow and without adding a magnet (control) for different capsule sizes, technologies for the introduction of magnetic nanoparticles (adsorption onto the shell and freezing), the number of magnetite nanoparticles introduced by freezing.

## Abbreviations

The following abbreviations are used in this manuscript:

RBCs	red blood cells
WBCs	white blood cells
LbL	layer-by-layer
MNPs	magnetic nanoparticles
FIL	freezing-induced loading
Parg	poly-L-arginine hydrochloride
DS	dextran sulfate sodium salt
TEM	transmission electron microscopy
SEM	scanning electron microscopy
DLS	dynamic light scattering
BSA	bovine serum albumin
FITC	fluorescein isothiocyanate
PBS	phosphate buffered saline
DBPS	Dulbecco’s modified phosphate buffered saline
NPs	nanoparticles
FBS	fetal bovine serum
EDTA	ethylenediaminetetraacetic acid
ROI	region of interest
OD	optical density
DMEM	Dulbecco’s modified Eagle medium
TRITC	tetramethylrhodamine
