# Supplementary figures and images for: Effect of Size on Magnetic Polyelectrolyte Microcapsules Behavior: Biodistribution, Circulation Time, Interactions with Blood Cells and Immune System
Source: Pharmaceutics. 2021 Dec 14;13(12):2147. doi: 10.3390/pharmaceutics13122147 (PMC8703762; doi:10.3390/pharmaceutics13122147)

## 1 $\mu\text{m}$ capsules

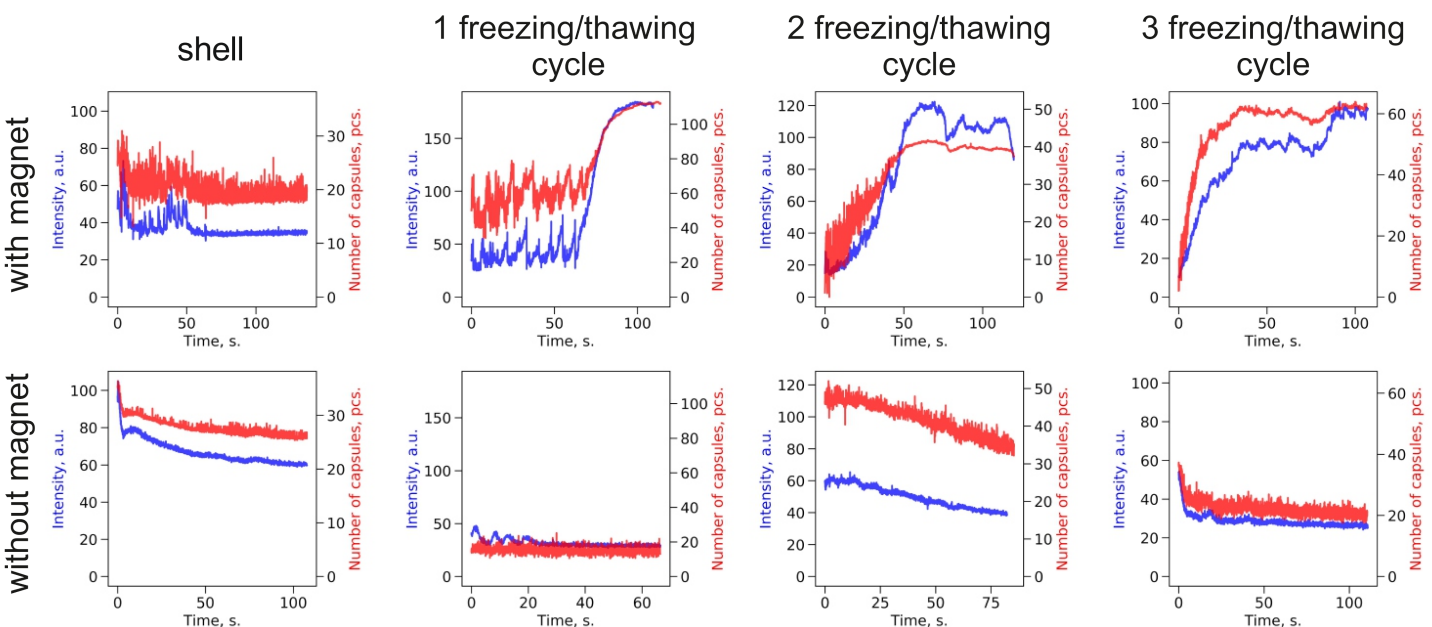

## 2.7 $\mu\text{m}$ capsules

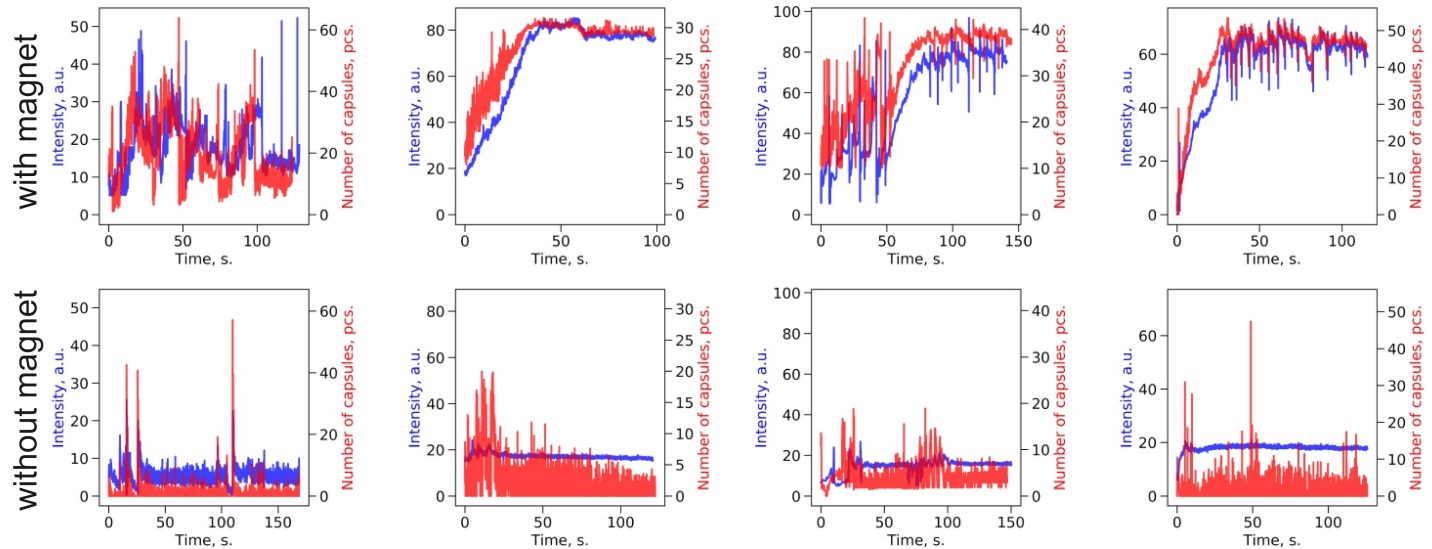

## 5.5 $\mu\text{m}$ capsules

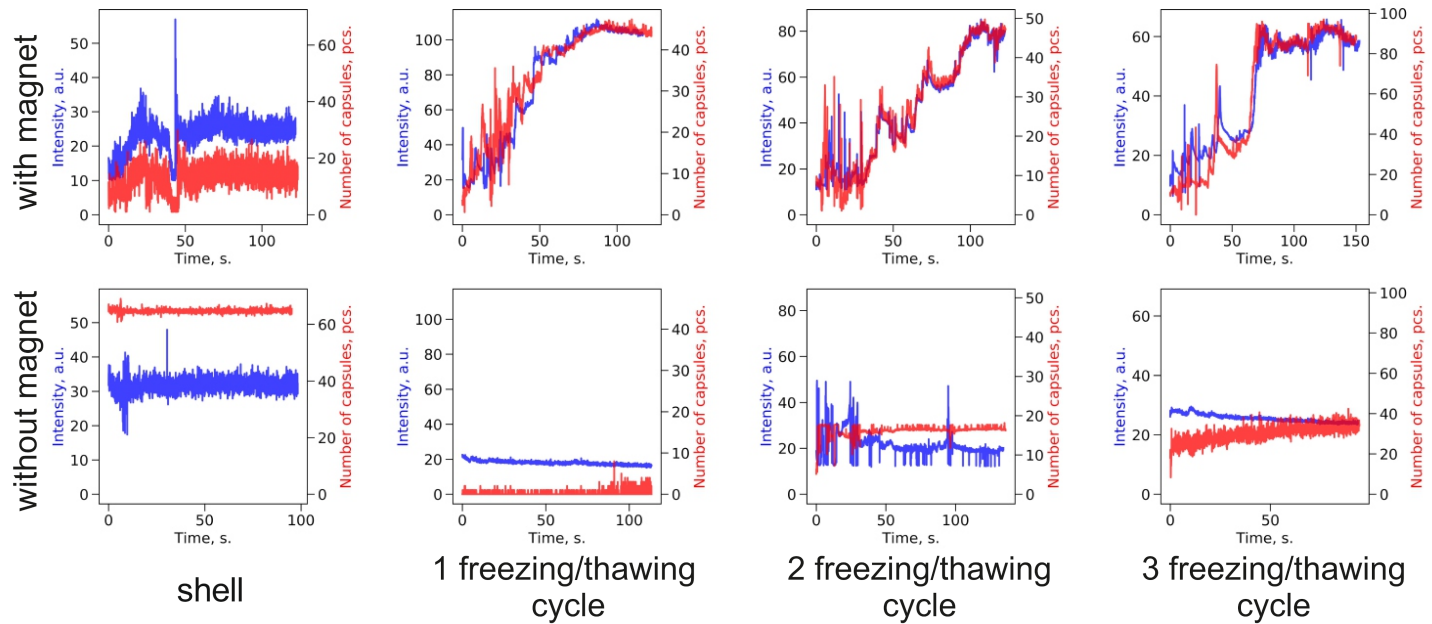

Supplement: Supplementary file 1 [file pharmaceutics-13-02147-s001.zip › Fig. S1.pdf]

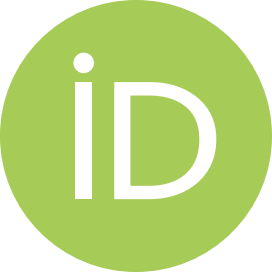

Supplement: Supplementary file 1 [file pharmaceutics-13-02147-s001.zip › Definitions/logo-orcid-eps-converted-to.pdf]

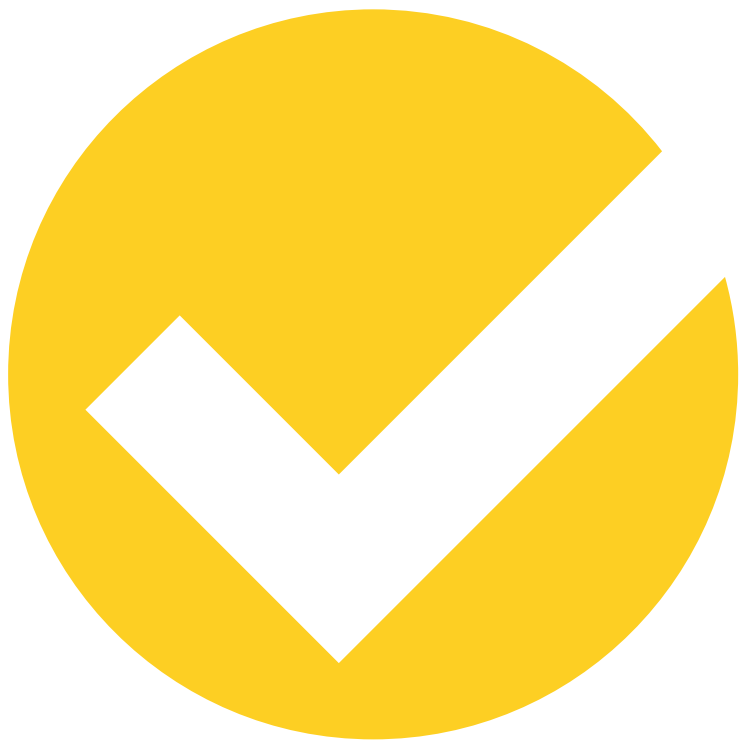

check for  
updates

Supplement: Supplementary file 1 [file pharmaceutics-13-02147-s001.zip › Definitions/logo-updates.pdf]
